# Attenuated Lower Limb Stretch-Shorten-Cycle Capacity in ACL Injured vs. Non-Injured Female Alpine Ski Racers: Not Just a Matter of Between-Limb Asymmetry

**DOI:** 10.3389/fspor.2022.853701

**Published:** 2022-03-31

**Authors:** Matthew J. Jordan, Nathaniel Morris, Sophia Nimphius, Per Aagaard, Walter Herzog

**Affiliations:** ^1^Canadian Sport Institute Calgary, Calgary, AB, Canada; ^2^Faculty of Kinesiology, The University of Calgary, Calgary, AB, Canada; ^3^School of Medical and Health Science, Centre for Human Performance, Edith Cowan University, Joondalup, WA, Australia; ^4^Department of Sports Science and Clinical Biomechanics, University of Southern Denmark, Odense, Denmark

**Keywords:** return to sport (RTS), jump asymmetry, plyometric, knee injuries, athlete monitoring, countermovement jump, CMJ

## Abstract

A retrospective analysis of routine countermovement jump (CMJ) testing, a coupled eccentric-concentric (stretch-shorten-cycle: SSC) movement, was performed in female elite alpine skiers with anterior cruciate ligament (ACL) reconstruction (ACLR) and without ACLR. A total of 567 tests obtained from the daily training environment were analyzed in 41 elite female athletes (non-injured control: *n* = 30, ACLR: *n* = 17), including *n* = 6 athletes with pre-injury data, between 16 and 32 years of age from alpine ski racing (*n* = 32) and skier cross (*n* = 9). Bilateral CMJ testing was conducted on a dual force plate system, and the limb-specific vertical ground reaction force (Fz) was analyzed to obtain the net eccentric deceleration impulse (Ecc), lower limb stiffness (Stiff), maximal vertical jump height (JH), peak external mechanical power (PP) exerted on the body center of mass (BCM), modified-reactive-strength-index (RSImod), and the loss in BCM velocity during the final phase of the takeoff Δ(Vmax–Vtakeoff). Eccentric and concentric phase-specific between-limb asymmetry indexes (AIs) were also calculated. Additive mixed effects models (AMMs) were used to compare the age-dependent and post-injury time course change between groups. The mean values for non-injured controls >25 years of age were used as a comparative benchmark for recovery given the absence of pre-injury data. Net eccentric deceleration impulse increased and Δ(Vmax–Vtakeoff) decreased with age for the non-injured control group (*p* < 0.001) while between-limb AI (mean ± SD) fell between 1 ± 5% for the concentric phase and 3 ± 7% for the eccentric deceleration phase. Between-limb asymmetry became smaller in ACLR skiers with time-from-surgery to reach non-injured control values by 2 years, but SSC function, such as JH and PP, remained depressed up to 5 years post-surgery (*p* < 0.01), indicating impairments in SSC function. This highlights the importance of evaluating SSC performance capacity alongside vertical jump force-time asymmetries in female ACLR alpine skiers.

## Introduction

There is a paucity of scientific literature on female elite alpine ski racers. Alpine skiers require high levels of lower body maximal muscle strength (Berg et al., 1995; Meyer et al., 2004; Raschner et al., 2012; Jordan et al., 2015b; Jordan et al., 2018) to maximize their speed in the descent using high-force eccentric and quasi-isometric muscle actions (Berg et al., 1995). Reports exist on sport-specific (Granan and Inacio, 2013) and sex-specific knee injury risk factors (Raschner et al., 2012; Bere et al., 2014; Steidl-Müller et al., 2018). For example, alpine ski racers are at high risk for knee injuries, especially anterior cruciate ligament (ACL) rupture, and ACL reinjury occurs frequently (Flørenes et al., 2009; Bere et al., 2014; Jordan et al., 2017a,c). ACL injuries in alpine ski racing typically occur due to high energy injury mechanisms during eccentric (braking) movements, such as landing from a jump (Bere et al., 2011). To address the prevalence of ACL injury and reinjury among alpine skiers, expansive neuromuscular testing and monitoring have been suggested to identify trainable neuromuscular deficits, such as abnormally high lower limb strength asymmetries before and after ACL injury (Raschner et al., 2012; Jordan et al., 2015a,b; Steidl-Müller et al., 2018; Jordan et al., 2020b).

The countermovement jump (CMJ) is a coupled eccentric-concentric movement that is typically classified as a slow stretch-shorten-cycle (SSC) action (i.e., ground contact time > 250 ms), is a principal neuromuscular test used alongside fast-SSC tests like the drop jump to evaluate lower limb mechanical muscle function (Caserotti et al., 2001, 2008; Thorlund et al., 2008; Jakobsen et al., 2012; Holsgaard-Larsen et al., 2014; Jordan et al., 2015a; Bishop et al., 2021). Vertical jump force-time analysis of the jumping athlete alongside measurement of the between-limb asymmetry index (AI) using a dual force plate system has become more commonplace in the context of ACL injury prevention, ACL rehabilitation, and performance evaluation (Thorlund et al., 2008; Holsgaard-Larsen et al., 2014; Gathercole et al., 2015a,b; Jordan et al., 2015a, 2018, 2020a,b; Bishop et al., 2019; Hart et al., 2019; Read et al., 2020, 2021). Further, it is clear that the eccentric deceleration phase (reversal of the downward acceleration of the body center of mass—BCM) and concentric phase need to be evaluated separately in athletes with ACL reconstruction surgery (ACLR) in the post-injury recovery period (Holsgaard-Larsen et al., 2014; Jordan et al., 2015a; Baumgart et al., 2017a; Hart et al., 2019; Read et al., 2020), and that between-limb asymmetries in these phases may differ depending on the surgical technique employed (Miles et al., 2019a; Jordan et al., 2022). Research examining the utility of the CMJ to monitor recovery in athletes with ACLR has found persistent deficits in both the eccentric and concentric phases. Not only can measures of vertical jump propulsive performance [e.g., jump height (JH), system peak external mechanical power] be obtained but also variables associated with SSC capacity and vertical jump strategy, such as eccentric deceleration kinetics, modified-reactive-strength-index (RSImod), rate of force development (RFD), and lower limb stiffness (Jakobsen et al., 2012; Holsgaard-Larsen et al., 2014; Jordan et al., 2015a; Bishop et al., 2021). Such measures have been used routinely as a component of longitudinal athlete monitoring, in lower body mechanical muscle testing, and to evaluate athlete recovery after ACL injury (Gathercole et al., 2015a,b; Jordan et al., 2015a, 2020b, 2022; Hart et al., 2019; Taberner et al., 2020; Read et al., 2021). For example, RSI measured in the drop jump was recently shown to predict the future risk of ACL reinjury after ACLR in competitive athletes (King et al., 2021).

In contrast, no knowledge exists about the time-course development of SSC capacity in female non-injured elite alpine skiers along with the subsequent impairment and recovery of SSC function after an ACL injury. This is crucial given that ACL injury risk changes with age in female skiers with a peak incidence occurring in the mid-teens (Raschner et al., 2012). A better understanding of the time-course change in SSC capacity can help sports performance and sports medicine practitioners more clearly understand how female alpine skiers progress as they age, and provide important normative data to guide long-term athlete development and ensure a safer post-injury return to sport. Thus, the purpose of this study was 2-fold. First, a non-linear analysis was conducted to evaluate the time-course change in SSC capacity in non-injured female alpine ski racers between 16 and 32 years of age. Second, we evaluated the recovery of SSC function after ACL injury and ACLR that included recovery of the eccentric deceleration and concentric kinetic impulse, respectively, as manifested by temporal change in absolute SSC function and the between-limb kinetic impulse AI.

## Materials and Methods

### Participant Characteristics

Repeated CMJ test data obtained from routine monitoring in the daily training environment were collected in 41 elite female athletes between 16 and 32 years of age from alpine ski racing (*n* = 32) and skier cross (*n* = 9; Table 1). The experimental protocols were approved by the University of Calgary Conjoint Health and Research Ethics Board (REB14-2270 and REB15-1094), and participants provided their written informed consent. Age-matched participants with no history of ACL injury (non-injured controls) were included along with pre-injury data of the ACLR group (*n* = 6) when available for the age-dependent analysis. Participants with a primary diagnosis of ACL rupture and subsequent ACLR were included for the post-injury time-dependent analysis. All ACLR athletes received semitendinosus tendon autografts. Participants with a history of lower limb injury that required surgery (e.g., leg fracture), osteoarthritis, or meniscal injury were excluded.

**Table 1:** Breakdown of measurement count and sample size by sport (countermovement jump–CMJ, anterior cruciate ligament reconstruction–ACLR, non-injured controls, min:max–the minimum and maximum number of CMJ tests *per* athlete).

	**Control**	**ACLR**
	**Count (** ***n*** **)**	
Total number of athletes (*n* with pre-injury data)	30 (6)	17
CMJ assessments (pre-injury test sessions)	442 (89)	125
Mean measurements/athlete (min:max)	16 (1:78)	8(1:20)
Standard deviation of measurements/athlete	21	6
Alpine skiing (*n* with pre-injury data)	22 (3)	13
Skier cross (*n* with pre-injury data)	8 (3)	4

### CMJ Testing Protocol

**Countermovement jump** force-time assessments were performed on a dual force plate system (Jordan et al., 2015a, 2017b, 2018). Following a standardized warm-up that included 10 min of light ergometer cycling and dynamic stretching for the lower body, participants performed 5 maximal effort CMJs with each jump repetition separated by 3 s of quiet standing. Participants were given a strong verbal encouragement from a certified exercise practitioner to maximize their vertical JH. CMJs were performed with the hands placed firmly on the hips. No other constraints on the jump strategy were made, and participants were allowed to choose a self-selected countermovement depth (Pérez-Castilla et al., 2021).

A detailed description of the vertical jump testing protocol and force-time analysis procedure has been reported previously (Jordan et al., 2015a, 2017b, 2018, 2020a). Briefly, the vertical ground reaction force (Fz) from the right and left legs was measured synchronously using a dual force plate system (AMTI AccuPower Force Platform, Watertown, MA, USA) at a sampling frequency of 1,500 Hz and recorded on a personal computer (MyoResearch Version 3.8, Noraxon, Scottsdale, AZ, USA). Data were exported and analyzed using a custom-built computer program (MATLAB R 2018b, MathWorks, Natwick, MA, USA). The velocity of the BCM was obtained by time integration of the instantaneous acceleration signal ($BCM\;Acceleration\;=\;\frac{Fz}{Body\;Mass}-9.81\;m/s^{2}$) calculated from the total Fz (right + left limb). Distinct CMJ jump phases were identified based on the velocity of the BCM. The eccentric deceleration phase was defined as the time interval between the maximum downward negative velocity to the point of zero velocity achieved at the initiation of the ascent (deepest BCM position). The concentric phase was defined from the deepest BCM position (zero BCM velocity) to the instant of jump takeoff (Fz = 0 N). The total impulse produced during the eccentric deceleration and concentric phases, respectively, was calculated by time integration of Fz over the selected time intervals (Jordan et al., 2015a, 2018, 2020a).

External mechanical power exerted on BCM was derived continuously throughout the jumping movement by calculating the instantaneous product of Fz and BCM velocity. JH was determined from the BCM vertical velocity at the instant of ground toe-off ($Jump\;Height=\frac{{Takeoff\;Velocity}^{2}}{2\times 9.81m/s^{2}}$). Representing the capacity to couple rapid knee extension and plantar flexion at the termination of the vertical jump, the numerical difference between maximum BCM velocity (Vmax) during the concentric (ascending) phase and the takeoff velocity (Vtakeoff) was determined Δ(Vmax–Vtakeoff) (Caserotti et al., 2001). The net impulse and lower limb stiffness in the eccentric deceleration phase ($Stiffness=\frac{\;\Delta Fz}{\Delta \;BCM\;Displacement}$) were determined to provide an assessment of the CMJ braking strategy (Jakobsen et al., 2012; Jordan et al., 2018). The BCM displacement was derived by double integration of the acceleration signal obtained from Fz (Jakobsen et al., 2012; Jordan et al., 2018). Finally, the duration of the jump takeoff (jump contraction time) was obtained from the start of the jump (BCM starting to move downward) to the instant of ground toe-off (Fz = 0 N) to determine the RSImod, calculated as:


(1) $$\begin{array}{r} RSImod=\left(\begin{array}{c} \frac{Flight\;Time\;of\;the\;BCM\;Derived\;from\;the\;Takeoff\;Velocity}{Jump\;Contraction\;Time} \end{array}\right) \end{array}$$

Modified-reactive-strength-index is typically calculated as $RSImod=\frac{Jump\;Height}{Contraction\;Time}\;$(Pérez-Castilla et al., 2021). However, in the present study, the vertical JH (obtained from the impulse-momentum method) was converted into the predicted flight time of the BCM, a measure that has also been referred to as the reactive strength ratio and is an accepted method for quantifying reactive strength capacity (Healy et al., 2018). A comparison of the phase-specific impulse produced by each limb was done using a between-limb AI calculation defined as:


(2) $$\begin{array}{r} AI\;=\left(\begin{array}{c} \frac{Right\;Limb\;Impulse-Left\;Limb\;Impulse}{Maximum\;Impulse\;of\;Left\;vs.\;Right\;Limb} \end{array}\right)*100 \end{array}$$

The best (highest) jump of the 5-CMJ was used in the statistical analysis for the vertical jump performance measures (i.e., JH and peak external mechanical power—measures of maximal jumping capacity) whereas a 5-jump-average was used for all other measures [i.e., RSImod, lower limb stiffness, net eccentric deceleration impulse, Δ(Vmax–Vtakeoff)] and the between-limb force-time AI calculations (concentric phase asymmetry, eccentric deceleration phase asymmetry). Peak external mechanical power, net eccentric deceleration impulse, and lower limb stiffness were normalized to body mass for the statistical analysis. Coefficients of variation for the outcome measures were as follows: (1) takeoff velocity = 1.96%; (2) flight time of the BCM calculated from the takeoff velocity = 1.96%; (3) maximum velocity of the BCM = 1.72%; (4) peak external mechanical power = 2.14%; (5) JH = 3.96%; (6) net eccentric deceleration impulse = 4.52%; (7) RSImod = 10.08%; (8) jump contraction time = 10.30%; and (9) lower limb stiffness = 14.11%.

### Statistical Analysis

To assess the time-course change in SSC function, separate additive mixed effects models (AMMs) were fitted for each of the jump variables with a smooth term for the age of the athlete (measured in years) and random intercepts for athlete. For the ACLR group, a smooth term for time-from-surgery was fit with random intercepts for athlete. AMMs were chosen to capture the complexity in the adaptive process and the potential for non-linear behavior in the outcome measures over time. Model diagnostics were performed to assess the fit of the models. Jump outcome variables were grouped into concentric phase variables [i.e., JH, peak external mechanical power, Δ(Vmax–Vtakeoff)] and eccentric deceleration phase variables (i.e., net eccentric deceleration impulse, lower limb stiffness, and RSImod) in all figures and tables. Finally, a linear mixed effects model was fit to assess the effects of group status and Δ(Vmax–Vtakeoff) on JH with random intercepts for athlete. All statistical analyses were done in R Studio, using the mgcv package to create the AMMs, the itsadug and ggplot2 packages to generate figures, and the lme4 package for the linear mixed effect model (R, Version 4.03). Statistical significance was set at α = 0.05 (two-tailed).

## Results

Model results for non-injured controls and ACLR athletes are presented in Tables 2, 3, respectively. For non-injured controls, the models explained between 72% (RSImod) and 90% (JH) of the deviance in the outcome measures. The deviances explained by the models for the eccentric deceleration phase in non-injured athletes were between 67% (lower limb stiffness) and 73% (net eccentric deceleration impulse).

**Table 2:** Model results for the non-injured controls vs. chronological age.

**Jump variable**	**Model**	***R^**2**^***	**Deviance explained %**	**Smooth terms**	**Significance of smooth terms**
Net eccentric deceleration impulse	Control	0.71	73.0	Age at test (years)	^***^
Lower limb stiffness	Control	0.65	67.3	Age at test (years)	^***^
Jump height	Control	0.89	89.9	Age at test (years)	^*^
Peak external mechanical power	Control	0.88	88.5	Age at test (years)	^***^
Modified reactive strength index	Control	0.69	71.5	Age at test (years)	^***^
Δ(Vmax-Vtakeoff)	Control	0.77	77.9	Age at test (years)	^***^
Eccentric impulse asymmetry	Control	0.44	46.7	Age at test (years)	^***^
Concentric impulse asymmetry	Control	0.54	56.3	Age at test (years)	^***^

The model formulas used to fit each model were developed in R via the bam function in the mgcv package. Significant differences at p < 0.05 and p < 0.001 indicated by ^*^ and ^***^ respectively.

*bam(Jump_Measure_~ s(Athlete_Age_)+ s(Athlete, bs = “re”), method = “REML”)*.

**Table 3:** Model parameters for the anterior cruciate ligament reconstructed (ACLR) group vs. the time from surgery.

**Jump variable**	**Model**	***R^**2**^***	**Deviance explained**	**Smooth terms**	**Significance of smooth terms**
Net eccentric deceleration impulse	ACLR	0.75	78.0%	Time from surgery (days)	^***^
Lower limb stiffness	ACLR	0.46	50.0%	Time from surgery (days)	
Jump height	ACLR	0.72	75.4%	Time from surgery (days)	^***^
Peak external mechanical power	ACLR	0.73	76.4%	Time from surgery (days)	^**^
Modified reactive strength index	ACLR	0.69	72.4%	Time from surgery (days)	^***^
Δ(Vmax-Vtakeoff)	ACLR	0.42	48.0%	Time from surgery (days)	
Eccentric impulse asymmetry	ACLR	0.56	60.7%	Time from surgery (days)	^***^
Concentric impulse asymmetry	ACLR	0.72	75.2%	Time from surgery (days)	^***^

The model formulas used to fit each model were developed in R via the bam function in the mgcv package. Significant differences at p < 0.01 and p < 0.001 indicated by **and *** respectively.

*bam(Jump_Measure ~ s(Time_from_Surgery) + s(Athlete, bs = “re”), method = “REML”)*.

For the ACLR group, the models explained between 48% Δ(Vmax–Vtakeoff) and 76% (peak external mechanical power) of the deviance in the outcome measures for the concentric phase and between 50% (lower limb stiffness) and 78% (net eccentric deceleration impulse) for the eccentric phase (Table 3).

**Figure 1:**
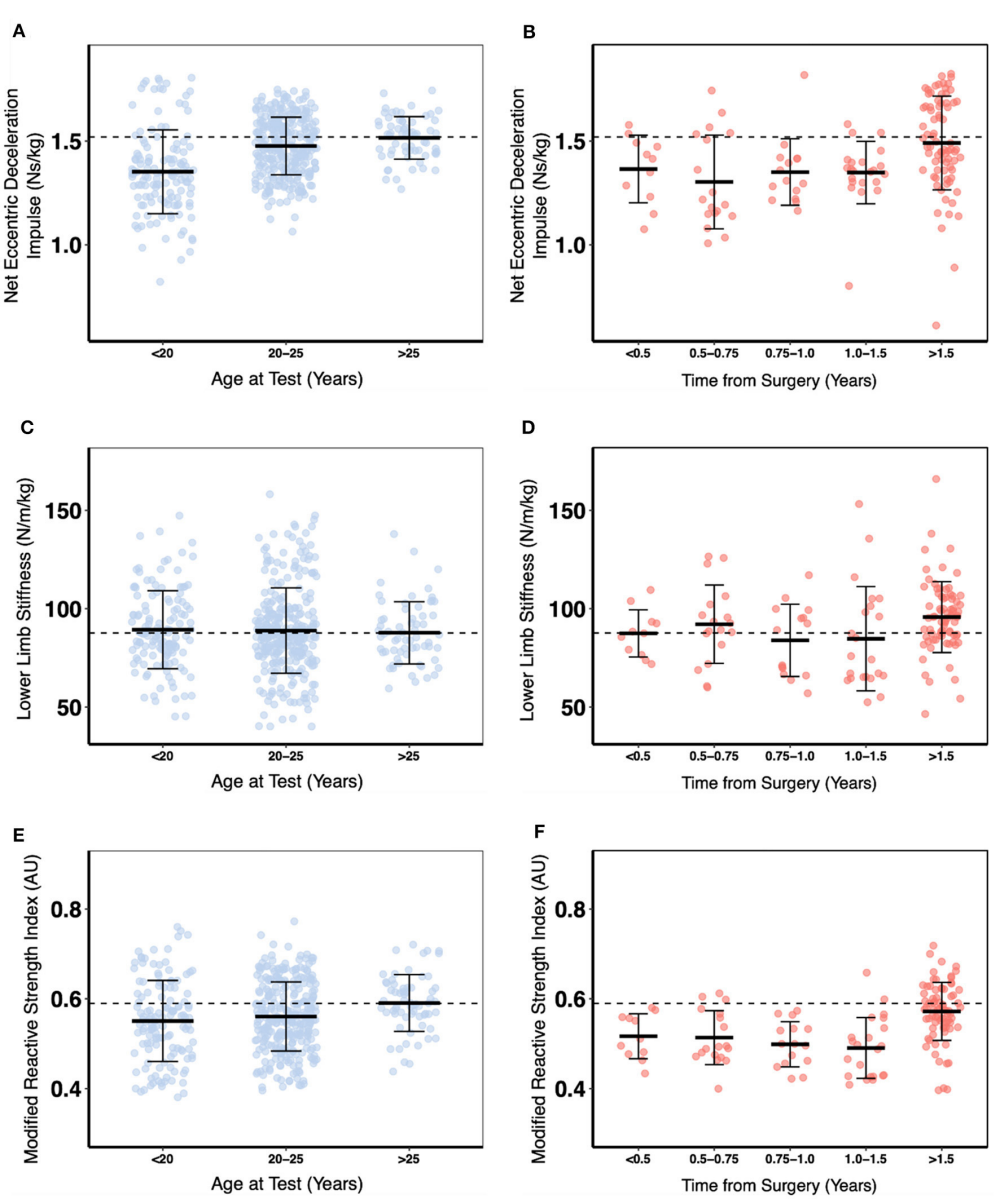
A descriptive comparison of eccentric deceleration phase measures and modified reactive strength index for non-injured controls vs. age and anterior cruciate ligament reconstructed (ACLR) skiers vs. time from surgery. The black dashed line indicates the mean value for non-injured controls > 25 years of age. **(A)** Net eccentric deceleration impulse control. **(B)** Net eccentric deceleration impulse ACLR. **(C)** Lower limb stiffness control. **(D)** Lower limb stiffness ACLR. **(E)** Modified reactive strength index control. **(F)** Modified reactive strength index ACLR.

**Figure 2:**
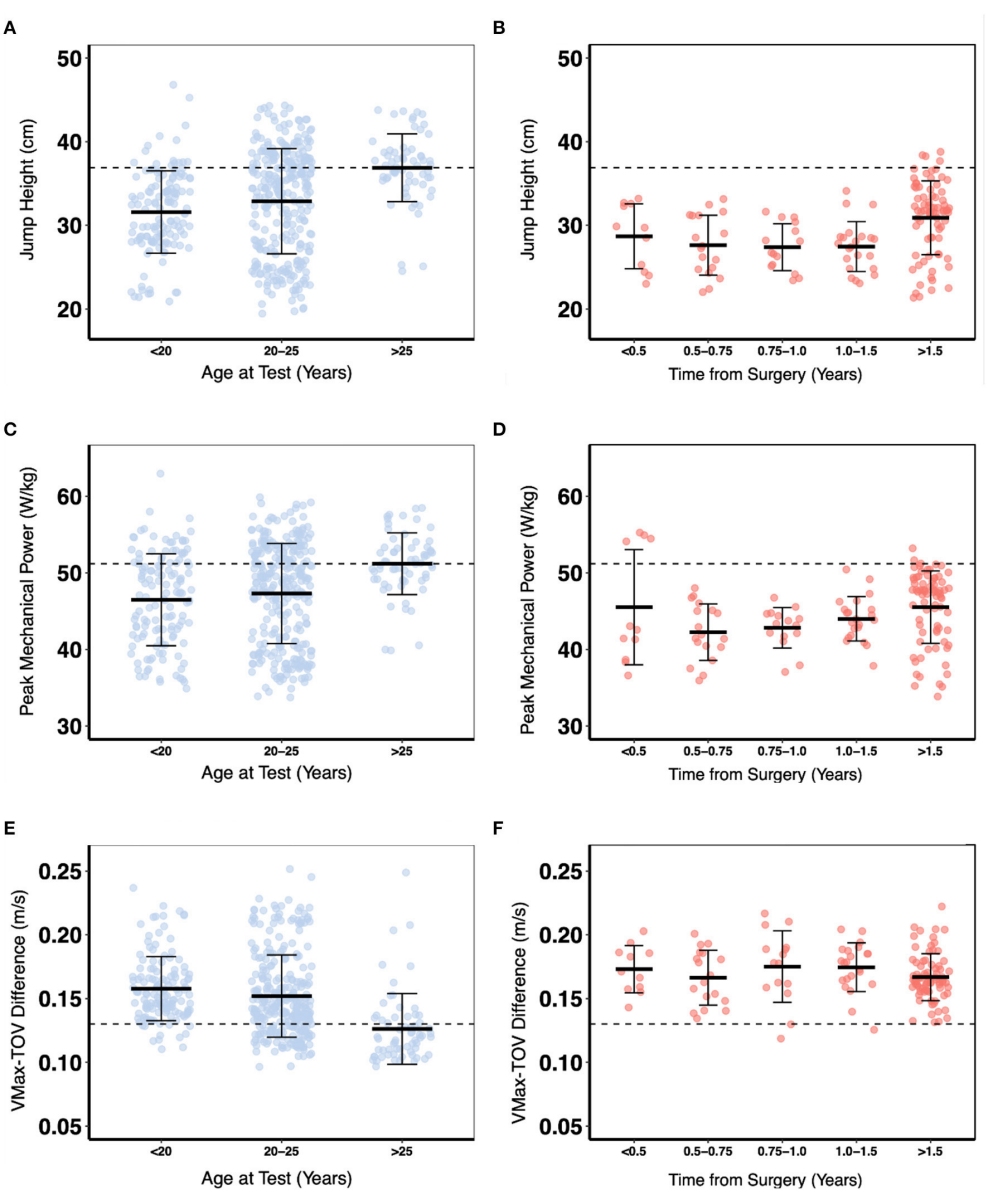
A descriptive comparison of concentric phase measures for non-injured control vs. age and anterior cruciate ligament reconstructed (ACLR) skiers vs. time from surgery. The black dashed line indicates the mean value for non-injured controls >25 years of age. Vmax-TOV Difference–Δ maximum velocity of the body center of mass and takeoff velocity. **(A)** Jump height control. **(B)** Jump height ACLR. **(C)** Peak mechanical power control. **(D)** Peak mechanical power ACLR. **(E)** Max velocity-takeoff velocity difference control. **(F)** Max velocity-takeoff velocity difference ACLR.

A descriptive analysis of lower limb SSC function by age-group (non-injured controls) and time-from-surgery (ACLR group) was conducted to establish normative benchmark data (defined here as the group mean of non-injured controls > 25 years of age). These data are presented in Figures 1, 2.

**Figure 3:**
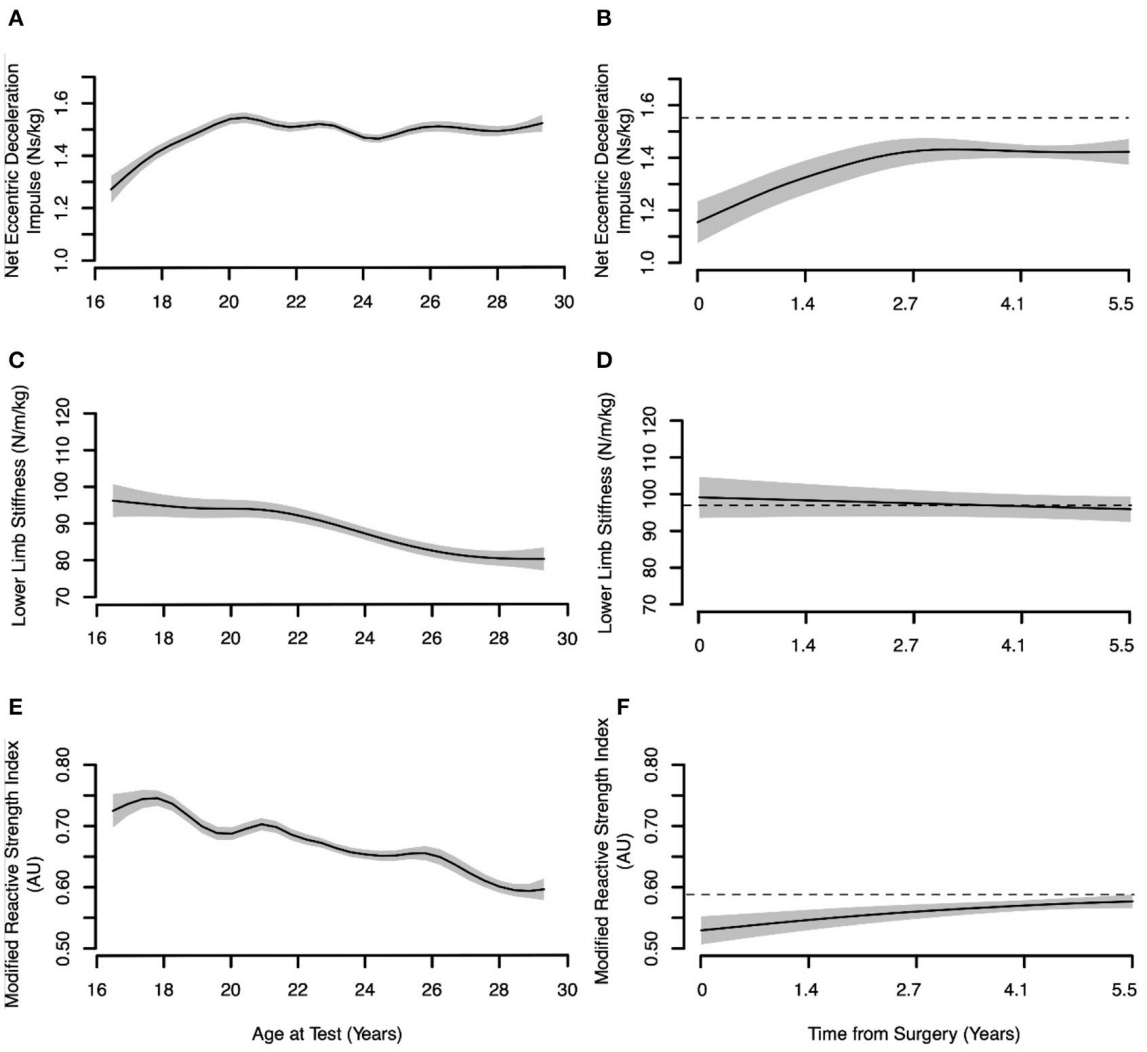
Age-dependent changes in eccentric deceleration phase jump measures and modified reactive strength index for non-injured controls and the post-surgical time-dependent change for the anterior cruciate ligament reconstructed (ACLR) group. The dashed black line shows mean values for non-injured controls > 25 years of age. Solid black lines show the mean value for the typical athlete ± the 95% CI marked in gray shading. **(A)** Net eccentric deceleration impulse control. **(B)** Net eccentric deceleration impulse ACLR. **(C)** Lower limb stiffness control. **(D)** Lower limb stiffness ACLR. **(E)** Modified reactive strength index control. **(F)** Modified reactive strength index ACLR.

The net eccentric deceleration impulse increased between 16 and 20 years of age and then plateaued while lower limb stiffness and RSImod declined moderately with age for the non-injured controls (*p* < 0.001) (Figure 3). For the ACLR group, the net eccentric deceleration impulse and RSImod increased between 0- and 2.7-year post-surgery in ACLR but remained below control values for more than 4-years post-surgery.

**Figure 4:**
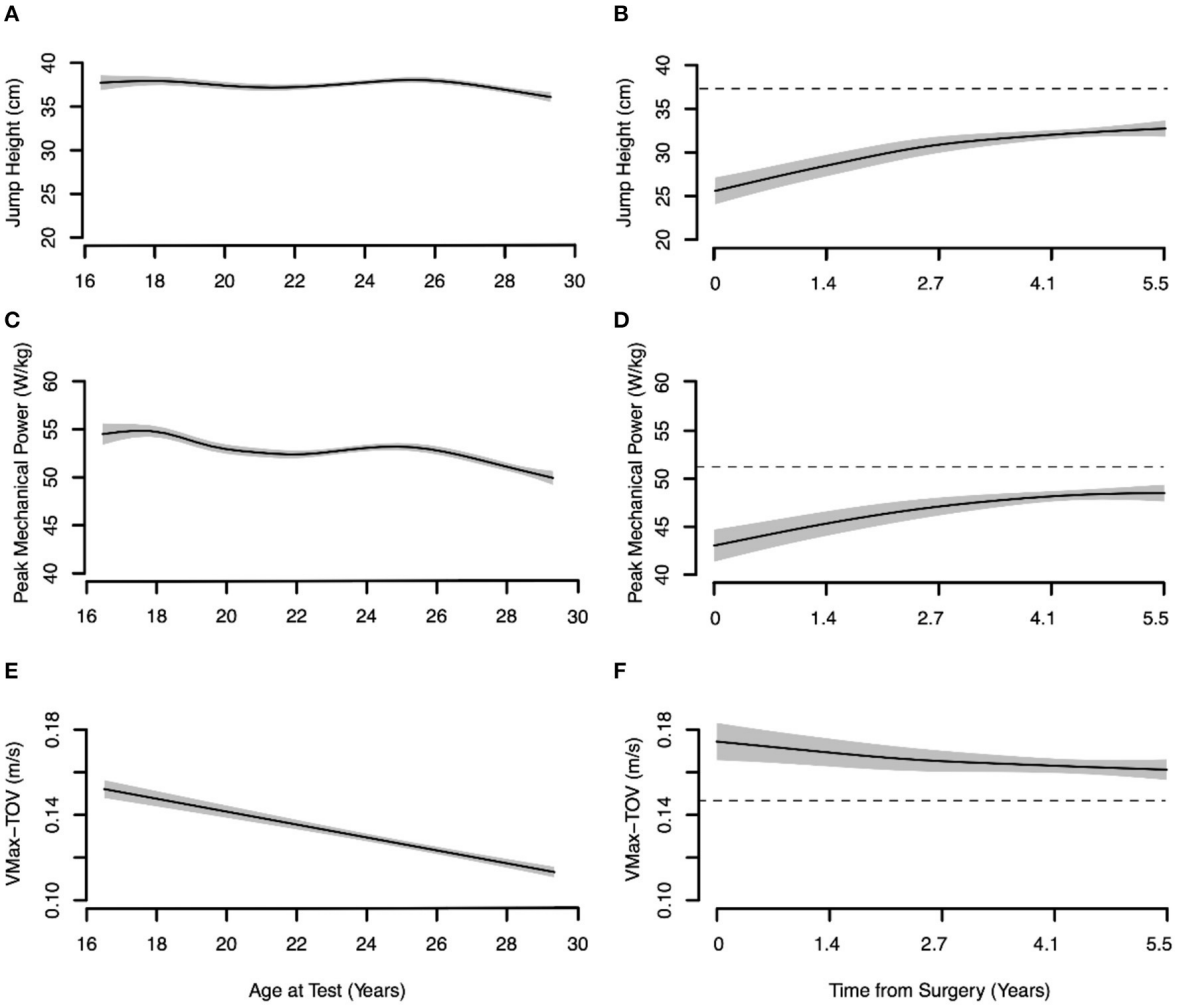
Age-dependent changes in concentric phase jump measures for the non-injured controls and post-surgical time-dependent changes in anterior cruciate ligament reconstructed (ACLR). Dashed black lines show mean value for non-injured controls > 25 years of age. Solid black lines show the mean value for the typical athlete ± the 95% CI marked in gray shading. Vmax-TOV Difference–Δ maximum velocity of the body center of mass and takeoff velocity. **(A)** Jump height control. **(B)** Jump height ACLR. **(C)** Peak mechanical power control. **(D)** Peak mechanical power ACLR. **(E)** VMax-TOV difference control. **(F)** VMax-TOV difference ACLR.

Vertical JH and peak external mechanical power declined moderately with increasing age for the non-injured controls whereas the Δ(Vmax–Vtakeoff) decreased linearly with increasing age in the non-injured controls (*p* < 0.001; Figure 4).

**Figure 5:**
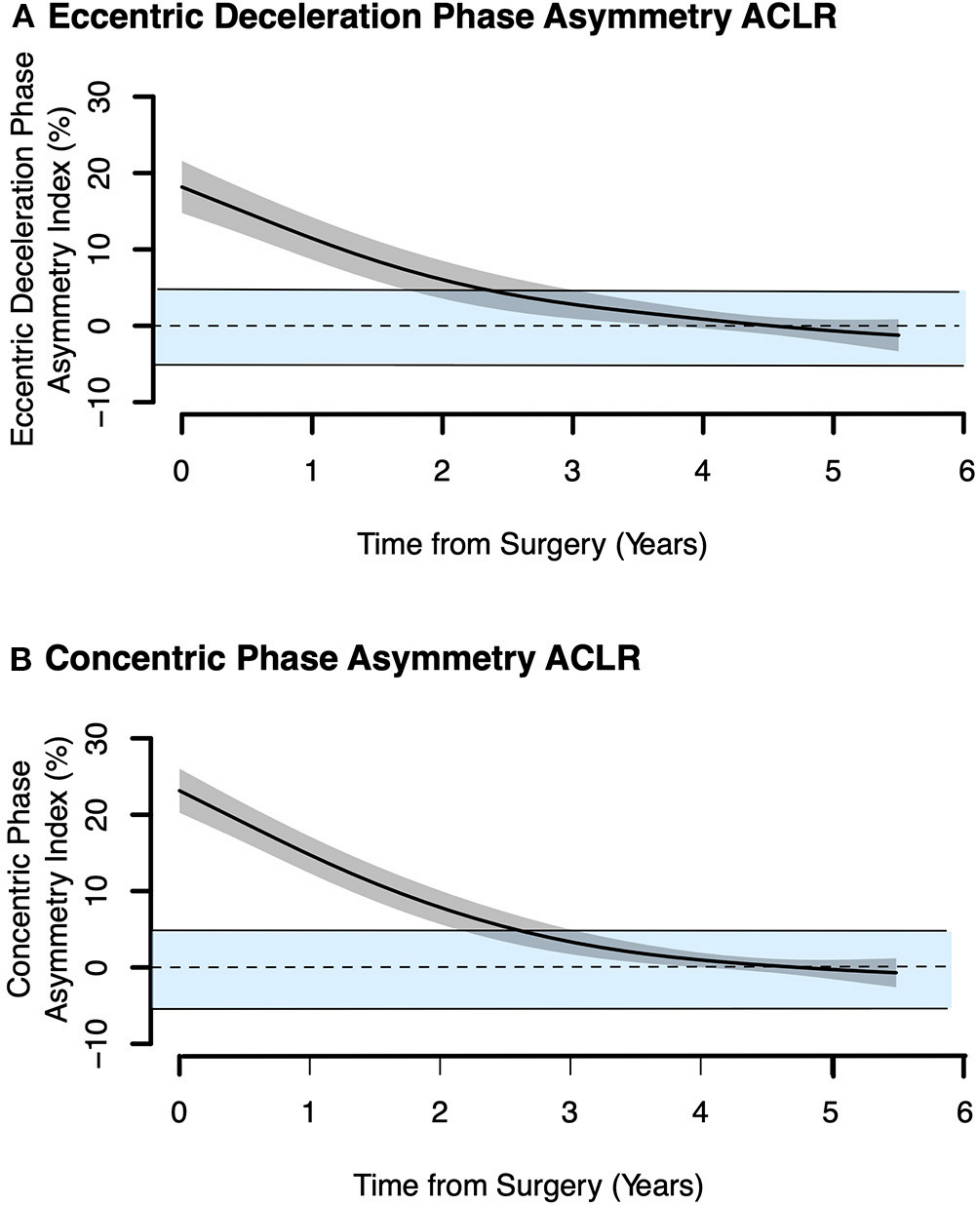
Between-limb asymmetry index for the the anterior cruciate ligament reconstructed (ACLR) group vs. time from surgery. Solid horizontal lines show ± 1 SD for non-injured controls, and 0% reference line (shaded blue region). Model output shows the mean value for the typical athlete ± the 95% CI marked in gray shading. **(A)** Eccentric deceleration phase asymmetry ACLR. **(B)** Concentric phase asymmetry ACLR.

Consistent with the changes in net eccentric deceleration impulse and RSImod, JH (*p* < 0.001) and peak external mechanical power (*p* < 0.01) increased for the ACLR group with time-from-surgery but plateaued after 2 years and did not return to the benchmark values (Figure 4). Finally, Δ(Vmax–Vtakeoff) showed only negligible changes with time-from-surgery and remained above the benchmark of the non-injured controls throughout the entire post-injury recovery period. As with the SSC outcome measures, it took more than 2 years for the between-limb AI of the ACLR group to approach non-injured control levels (Figure 5). Normative between-limb asymmetry data are provided in Table 4.

**Table 4:** Descriptive statistics for phase-specific between-limb asymmetry for the non-injured controls and anterior cruciate ligament reconstructed (ACLR) group stratified by time-since-surgery.

	**CMJ eccentric deceleration phase asymmetry (%)**			**CMJ concentric phase asymmetry (%)**		
**Group**	**Mean**	**SD**	**Range**	**Mean**	**SD**	**Range**
Control	3.0	7.7	(−26.3–35.0)	1.2	4.8	(−22.0–15.3)
**ACLR**
<0.5 y	0.4	7.2	(−10.8–9.6)	4.6	6.4	(−5.0–12.4)
0.5–0.75 y	6.0	12.0	(−13.9–42.8)	8.9	9.9	(−3.0–37.4)
0.75–1 y	4.5	7.6	(−13.5–17.8)	8.7	8.0	(−1.3–24.7)
1–1.5 y	4.9	4.9	(−7.6–13.2)	4.8	4.8	(0.4–19.6)
>1.5 y	−1.3	5.5	(−24.7–10.8)	5.6	5.6	(−23.0–14.7)

**Figure 6:**
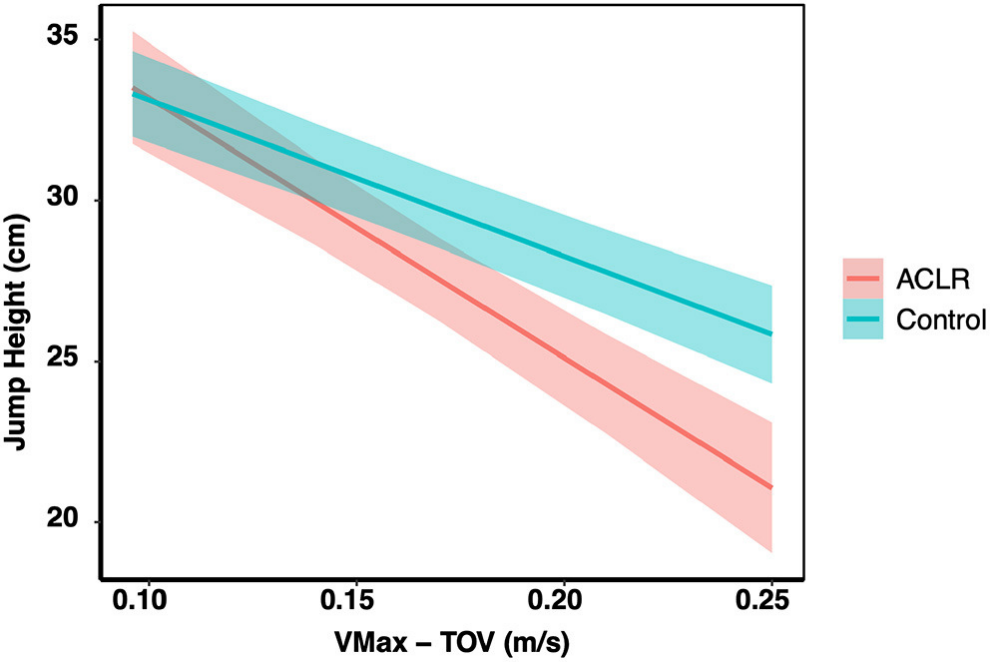
Relationship between group status (anterior cruciate ligament reconstructed–ACLR, non-injured control–Control), Vmax-TOV–Δ maximum velocity of the body center of mass and takeoff velocity and vertical jump height. A significant group x Vmax-TOV was found (*p* < 0.01). For every 0.1 m/s increase in the Vmax-TOV, jump height was decreased by 9.3 cm for the ACLR group and 4.9 cm for the Control group.

Finally, an interaction effect was found between Δ(Vmax–Vtakeoff) and injury status on JH (X^2^ = 9.4, *df* = 1, *p* < 0.01; Figure 6). As the Δ(Vmax–Vtakeoff) increased, JH decreased for the Control group (*t* = −10.1, *df* = 478.9, *p* < 0.0001) and the ACLR group (*t* = −8.7, *df* = 135.9, *p* < 0.0001). However, the effects of increasing Δ(Vmax–Vtakeoff) on JH were greater for the ACLR group. Here, for every 0.1 m/s increase in the Δ(Vmax–Vtakeoff), JH decreased by 9.3 cm compared to 4.9 cm for the non-injured control group.

## Discussion

This retrospective study evaluated the change in lower limb SSC capacity over a 16-year period for non-injured female ski racers and a 5-year post-surgical period for female alpine skiers with ACLR, to document the time-course of recovery after ACLR in this athlete population and the age-dependent changes in non-injured skiers. With increasing age, net eccentric deceleration impulse increased while RSImod and lower limb stiffness decreased for the non-injured controls. On average, between-limb asymmetries for the non-injured control were <5%. It took nearly 2 years for the between-limb asymmetries of the ACLR group to reach the value of the non-injured controls, but at 5 years post-surgery, SSC function remained depressed especially JH and peak external mechanical power, highlighting the importance of evaluating lower limb SSC capacity alongside measures of lower limb asymmetry when performing vertical jump force-time analysis.

Such analyses seem highly relevant given the risk of ACL injury and reinjury in alpine ski racing (Bere et al., 2014; Jordan et al., 2017a,c) and the long-term impact of ACL injury/reinjury on the knee joint health of skiers (Jordan et al., 2017c). For example, the relative risk of ACL injury amongst elite female ski racers is 1.2 ACL injuries *per* 1,000 runs, with female developmental level skiers demonstrating even greater (2.3-fold) relative risk for ACL injury compared to male skiers, and with nearly 30% of alpine ski racers sustaining ACL reinjury (Raschner et al., 2012; Bere et al., 2014; Jordan et al., 2017a,c). Further, in a previous retrospective analysis of ACL injuries amongst elite alpine ski racers, it was shown that a high fraction of athletes suffered serious concomitant injuries that include complex meniscal tears and severe articular cartilage injury, while a significant proportion of skiers with ACLR experienced subsequent surgeries that documented a substantial worsening of their knee joint health (Jordan et al., 2017c). Additionally, despite returning to sport and competition, the affected limb of ACLR ski racers demonstrated persistent muscle strength and power deficits compared to the contralateral limb and compared to the limbs of non-injured skiers (Jordan et al., 2015a,b; Jordan et al., 2017b). As neuromuscular deficits, such as reduced SSC capacity (Read et al., 2022), are associated with ACL reinjury (Grindem et al., 2016; King et al., 2021), continuous monitoring of neuromuscular function before and after ACL injury would seem essential for elite alpine skiers.

Indeed, the present data highlight the importance of monitoring lower limb mechanical muscle function, such as SSC capacity, in female alpine ski racers after ACLR. To this end, the models and data that are presented in the present study can help sport performance and sport medicine practitioners working with alpine ski racers to establish sport- and age-specific norms for guidance toward a safe return to sport after ACLR. An additional contribution of this work is the use of AMMs that account for non-linearities and complexity in biological systems (Bolker et al., 2009; Pedersen et al., 2019), as increasingly recommended in the sport performance and sports medicine scientific literature (Windt et al., 2018; Bailey, 2019; Fonseca et al., 2020). This is important in the management of return to sport and return to competition decision making after ACLR as the post-injury time period may be characterized by regression in neuromuscular function after an athlete has returned to sport and competition (Jordan et al., 2020b), and on average ACL reinjury occurs nearly 2 years after the first ACL injury in alpine ski racers (Jordan et al., 2017c). This suggests the need for expansive and frequent neuromuscular testing in ACL-injured athletes well-beyond when they return to sport, such as including routine neuromuscular monitoring in conjunction with discrete time point testing, which is often used as clearance criteria for return sport decision-making in athletes with ACLR (Grindem et al., 2016; Webster and Hewett, 2019; King et al., 2021).

There is limited scientific evidence that address the ideal frequency for monitoring SSC function in athletes recovering from ACLR. However, similar to monitoring the training process in non-injured athletes, practitioners may be interested in monitoring performance fatigability (i.e., the effects of fatigue on neuromuscular performance) (Halson, 2014; Enoka and Duchateau, 2016) and/or evaluating the training response (Smith, 2003). Here, a case study analysis of elite athletes recovering from ACLR used routine CMJ testing to monitor SSC function throughout the return to sport and return to competition transitions (Jordan et al., 2020b; Taberner et al., 2020). The impact of ACL injury on the non-affected contralateral limb must be considered as well, evidenced by the potential for marked contralateral limb decline in SSC function (Read et al., 2022), explosive strength (RFD) (Mirkov et al., 2017), and knee extensor strength more than 18 months after ACLR (Chung et al., 2015; Jordan et al., 2020b).

The statistical models employed in the present study showed that the net eccentric deceleration impulse increased in the non-injured skiers until just after 20 years of age at which point it plateaued. The finding of greater net eccentric deceleration impulse in older elite alpine ski racers compared to adolescent skiers has been documented elsewhere (Jordan et al., 2018). The net eccentric deceleration impulse is calculated in the time interval between the maximal downward BCM velocity and the deepest BCM position when the BCM velocity is zero (i.e., eccentric-concentric transition point). This value reflects the impulse required to bring the BCM momentum to zero prior to the ascent phase and is directly related to the downward velocity of the BCM through the impulse-momentum relationship. Here, a greater net eccentric deceleration impulse indicates a higher peak downward velocity of the BCM. The possibility that this jump strategy adaptation is unique to the long-term athlete development of alpine ski racers should be investigated further, especially given the preponderance of high-force eccentric/quasi-isometric muscle actions involved in alpine skiing (Berg et al., 1995). Additionally, the Δ(Vmax–Vtakeoff) was evaluated as a measure of neuromuscular coordination during the extension phase of the CMJ where the summation of the joint velocities can be assessed by the difference between the maximum concentric BCM velocity and BCM velocity at the instant of takeoff (Caserotti et al., 2001, 2008). A significant optimization (minimization) of the difference between maximum concentric BCM velocity and BCM velocity at the instant of takeoff Δ(Vmax–Vtakeoff) was also noted in non-injured alpine skiers with increasing age. Finally, consistent with previous findings, between-limb asymmetry in the eccentric deceleration and concentric phases of the CMJ were small in the non-injured alpine skiers (Jordan et al., 2015a, 2018).

The statistical models for ACLR athletes demonstrated substantial and persistent deficits in lower limb SSC capacity for several years after ACLR evidenced by a significant depression in the outcome measures >2 years post-surgery, and a plateau in CMJ function up to 5 years post-surgery where JH and peak external mechanical power remained below the benchmark value of elite non-injured alpine skiers >25 years of age. This benchmark included senior elite female alpine ski racers, such as World Cup medalists, and was determined to be a superior method for establishing post-ACLR recovery criteria compared to the frequent practice of using the strength capacity of the post-surgical non-injured contralateral limb of the ACLR athlete (Read et al., 2022). In contrast, between-limb AI in ACLR skiers was found to approach the level of non-injured skiers when compared at ~2 years post-surgery. The between-limb AI measured in a variety of strength tests is often used as the primary criteria to assess return to sport readiness but the efficacy of this approach has been questioned (Webster and Hewett, 2019). Given that ACL injury is known to cause contralateral limb strength impairments (Chung et al., 2015; Read et al., 2022), the utility of between-limb asymmetry measurements in jumping has been questioned recently (Grindem et al., 2016; King et al., 2021; Read et al., 2022). Notably, a more favorable limb AI may be achieved due to reduced strength capacity in both limbs, providing a false-positive indication of neuromuscular recovery.

Our study provides further evidence in support of this notion and illustrates the importance of considering not only between-limb asymmetry but also, overall SSC performance capacity expressed in absolute terms to account for the possibility of contralateral limb strength impairments. This was also emphasized in a recent case study evaluating the post-ACLR recovery of an elite female alpine ski racer based on pre- and post-injury data noting marked declines in contralateral limb knee extensor strength and RFD that occurred at 18 months post-surgery (Jordan et al., 2020b). SSC performance capacity in ACLR athletes has been put forward as a key component of expansive neuromuscular testing efforts following ACLR (Buckthorpe, 2019; Read et al., 2022), including CMJ testing where reduced capacity of the ACLR limb has been shown to persist more than 9 months post-surgery in professional soccer players (Read et al., 2020) and in elite alpine ski racers (Jordan et al., 2015a, 2018). The limitations of between-limb asymmetry testing were also illustrated recently where reduced leg spring stiffness and reactive strength but not between-limb asymmetry predicted the risk of ACL reinjury in high-level athletes (King et al., 2021). The negative impact of ACL injury on contralateral neuromuscular function may be especially apparent during ballistic movements, as manifested by reduced RFD in the leg extensors of the non-affected limb compared to healthy controls (Mirkov et al., 2017). Emerging evidence also underlines that the kinetic asymmetries measured in the unilateral (single leg) vertical jump and bilateral vertical jump cannot be equated (Baumgart et al., 2017a,b; Cohen et al., 2020), and that characteristics of unilateral vs. bilateral vertical jump ground reaction force asymmetries can differ depending on the International Knee Documentation Committee (IKDC) Subjective Form score (i.e., the patient's subjective rating of knee function after ACLR) (Baumgart et al., 2017a,b). Indeed, considerable variation in the CMJ force-time asymmetries was found for the ACLR group in the present investigation (*c.f*. Table 4). However, we did not document the IKDC score, and we are unable to present a detailed account of the pattern of combined injury. But previous studies on the nature of knee injuries in alpine ski racers diagnosed with a primary ACL tear indicate the potential for severe knee trauma and multi-ligament injury (Jordan et al., 2017c), which may account for the variation shown here and the possibility for prolonged neuromuscular recovery.

It has been suggested that the presence of compensatory movement strategies in ACLR athletes may be detectable in variations of the bilateral vertical jump (such as the CMJ) whereas unilateral jumping may provide a better indication of lower limb strength capacity (Cohen et al., 2020). To this end, not only did we evaluate JH and peak external mechanical power (measures of vertical jump performance) but also we assessed vertical jump strategy. Vertical JH, peak external mechanical power, and the net eccentric deceleration impulse remained substantially below benchmarks up to 5 years post-surgery. Conversely, RSImod demonstrated a near-complete recovery by 5 years and a minimal change in lower limb stiffness was found after ACLR. In the present study, RSImod was calculated as the ratio of the predicted flight time of the BCM obtained from the takeoff velocity divided by the jump contraction time but this method may lack sensitivity for detecting deficits in athletes recovering from ACLR, and a kinetic analysis of reactive strength capacity is recommended to address this concern (Read et al., 2022). Further, while lower limb stiffness has been measured in the CMJ to evaluate slow-SSC function in other investigations (Jakobsen et al., 2012; Jordan et al., 2018), it is typically assessed during fast SSC movements (ground contact times < 250 ms), such as the cyclic hop and vertical drop jumps (Komi and Bosco, 1978; Blickhan, 1989; Nicol et al., 2006). Here, the leg spring stiffness of the jumping human is characterized with respect to the energy lost in a fast SSC movement with task constraints to execute the jump as fast as possible while jumping as high as possible (Blickhan, 1989). Participants in our study were asked only to jump as high as possible with no constraint on the countermovement depth or speed of execution. Thus, the present finding of minimal change in lower limb stiffness after ACLR likely reflects a stable jump strategy rather than an indication of leg spring stiffness *per se*. Future research is required to examine the relationship between lower limb stiffness measured in the CMJ, leg spring stiffness in the drop jump and cyclic hop, and the best analytical approach to track recovery in athletes with ACLR.

Minimal recovery was observed for Δ(Vmax-TOV). The capacity to minimize the loss in BCM velocity at the instant of ground toe-off relative to the maximal BCM velocity reached during the takeoff phase [i.e., minimize Δ(Vmax–Vtakeoff)] has been used in other populations to identify impairments in neuromuscular function (Caserotti et al., 2001, 2008), but it has received little attention in athletes with an ACL injury. The specific neuromuscular factor(s) underpinning the long-term lack of recovery in Δ(Vmax–Vtakeoff) is (are) impossible to determine given the retrospective nature of the present study and any interpretation of the mechanisms potentially underlying these results are speculative. But surface electromyography (EMG) recordings from the quadriceps and hamstring muscle groups of ACLR elite alpine ski racers have shown decreased quadriceps muscle activity and increased hamstring muscle co-activity (protecting the reconstructed ACL) during the final takeoff phase in vertical jump testing compared to non-injured skiers (Jordan et al., 2017b). Not only might this distinct muscle activation pattern contribute to the increased between-limb asymmetry in takeoff kinetics observed in ACLR athletes, but it may also result in reduced BCM velocity during the final takeoff phase. Consequently, Δ(Vmax–Vtakeoff) would remain chronically increased in this athlete population, potentially reflecting a less effective energy transfer in the final phase of the vertical jump. Alternatively, the lack of recovery in Δ(Vmax–Vtakeoff) following ACLR may be caused by sustained impairments in plantar flexor muscle function (reduced strength, power, RFD) in both the affected and non-affected limbs, but this notion requires further scientific study. There appears to be limited data on the effects of ACL injury on ankle flexor function (strength, power, and RFD), and conflicting evidence to the ideas discussed here, namely, that ankle flexor strength may remain chronically impaired after ACLR (Thomas et al., 2013; Hoch et al., 2019). While pre-surgical ankle flexor strength deficits may be evident, it appears that ACLR patients can fully restore ankle flexor strength compared to the persistent deficits found for the knee joint musculature (Thomas et al., 2013). Nevertheless, reduced ankle flexor power in the affected limb of ACLR participants during the vertical jump has been reported previously (De Fontenay et al., 2014), alongside alterations in the neuromuscular control strategy of the hip, knee, and ankle joints during jump testing (Paterno et al., 2014; Blache et al., 2017).

It should be noted that the BCM velocity correlates strongly with vertical JH due to the linear change in the BCM velocity across the propulsion phase (Linthorne, 2020), and the fact that JH is determined directly from the vertical takeoff velocity according to Newton's principle of mechanical energy conservation (*E*_*potential*_+*E*_*kinetic*_ = *Constant*) during the flight phase of jumping. This may introduce spurious correlations between JH and measures that include the BCM velocity in the calculation [e.g., Δ(Vmax–Vtakeoff)] (Linthorne, 2020). While the BCM velocity increased linearly throughout the ascent phase to the instant of the maximum BCM velocity, it then declined linearly through to the point of takeoff. Notably, vertical JH and the Δ(Vmax–Vtakeoff) were inversely correlated, meaning that Δ(Vmax–Vtakeoff) minimized as JH increased, and a group × Δ(Vmax–Vtakeoff) interaction effect was found (*c.f*. Figure 6). Interestingly, an increase in the Δ(Vmax–Vtakeoff) resulted in a greater decline in JH for the ACLR group (9.3 cm) compared to the Control group (4.9 cm). Potential neuromuscular mechanisms underpinning these findings were previously discussed but more investigation is required to test these ideas. Given the current findings, the utility of the Δ(Vmax–Vtakeoff) as a mechanical biomarker of SSC recovery after ACLR should be explored further with respect to potential neuromuscular mechanisms (e.g., intermuscular coordination control strategies, joint-specific strength loss, protective mechanisms, such as increased hamstring coactivation, reduced voluntary activation) that might lead to better rehabilitation protocols to restore vertical jump capacity after the ACL injury.

## Limitations

As a limitation of the present study, the retrospective analysis of data obtained from the real-world training environment of elite athletes limited our capacity to control the measurement frequency, the overall sample size, and the lack of sample balance between groups. These limitations were partially accounted for using additive mixed effects modeling, which is robust against participant attrition. Nevertheless, the present data were collected in top-level, elite female athletes, and as such provide a unique perspective on the sport-specific neuromuscular capacities that include SSC function in female ski racers, and the subsequent impact and recovery of lower limb mechanical muscle function after ACLR. The generalizability of these results to other athlete populations is unknown but as the CMJ is routinely used in different populations after ACLR (Holsgaard-Larsen et al., 2014; Miles et al., 2019b; Read et al., 2020; Costley et al., 2021), our models can help sports performance practitioners estimate the time-course of recovery in SSC function after the ACL injury. Additionally, by analyzing lower limb SSC deficits using a phase-specific approach, such as in the present study, practitioners and clinicians may be able to devise targeted rehabilitation strategies to address eccentric deceleration, concentric, and terminal phase deficits during CMJ testing to restore vertical jump capacity.

## Conclusion

The present study highlights the importance of long-term athlete monitoring after ACL injury and emphasizes the importance of monitoring SSC performance capacity alongside measures of between-limb asymmetry in ACLR female ski racers. This study further highlights the relevance of including kinetic measures of vertical jump performance beyond vertical JH in female alpine ski racers, particularly, but not exclusively connected to the eccentric deceleration phase of the CMJ.

## Data Availability Statement

The datasets presented in this article are not readily available because the data is currently not available for inter-institution sharing according to our research ethics approval. Requests to access the datasets should be directed to mjordan@ucalgary.ca.

## Ethics Statement

The studies involving human participants were reviewed and approved by University of Calgary Conjoint Health and Research Ethics Board. Written informed consent to participate in this study was provided by the participants or the participants' legal guardian/next of kin.

## Author Contributions

MJ and NM performed the analysis and prepared the manuscript. SN, PA, and WH provided scientific input to the testing concepts, analysis, and interpretation along with manuscript editing. All authors contributed to the article and approved the submitted version.

## Funding

This study was supported in part by the Canada Research Chair Programme (WH) and the Killam Chair at the University of Calgary (WH), Mitacs Accelerate, Own the Podium and the Canadian Sport Institute Calgary.

## Conflict of Interest

The authors declare that the research was conducted in the absence of any commercial or financial relationships that could be construed as a potential conflict of interest.

## Publisher's Note

All claims expressed in this article are solely those of the authors and do not necessarily represent those of their affiliated organizations, or those of the publisher, the editors and the reviewers. Any product that may be evaluated in this article, or claim that may be made by its manufacturer, is not guaranteed or endorsed by the publisher.
